# High-fidelity correction of genomic uracil by human mismatch repair activities

**DOI:** 10.1186/1471-2199-9-94

**Published:** 2008-10-27

**Authors:** Erik D Larson, David W Bednarski, Nancy Maizels

**Affiliations:** 1 Department of Immunology, University of Washington School of Medicine, Seattle, WA, 98195-7650, USA; 2 Department of Biochemistry, University of Washington School of Medicine, Seattle, WA, 98195-7650, USA; 3 School of Biological Sciences, Illinois State University, Normal, IL, 61790-4120, USA

## Abstract

**Background:**

Deamination of cytosine to produce uracil is a common and potentially mutagenic lesion in genomic DNA. U•G mismatches occur spontaneously throughout the genome, where they are repaired by factors associated with the base excision repair pathway. U•G mismatches are also the initiating lesion in immunoglobulin gene diversification, where they undergo mutagenic processing by redundant pathways, one dependent upon uracil excision and the other upon mismatch recognition by MutSα. While UNG is well known to initiate repair of uracil in DNA, the ability of MutSα to direct correction of this base has not been directly demonstrated.

**Results:**

Using a biochemical assay for mismatch repair, we show that MutSα can promote efficient and faithful repair of U•G mismatches, but does not repair U•A pairs in DNA. This contrasts with UNG, which readily excises U opposite either A or G. Repair of U•G by MutSα depends upon DNA polymerase δ (pol δ), ATP, and proliferating cell nuclear antigen (PCNA), all properties of canonical mismatch repair.

**Conclusion:**

These results show that faithful repair of U•G can be carried out by either the mismatch repair or base excision repair pathways. Thus, the redundant functions of these pathways in immunoglobulin gene diversification reflect their redundant functions in faithful repair. Faithful repair by either pathway is comparably efficient, suggesting that mismatch repair and base excision repair share the task of faithful repair of genomic uracil.

## Background

Uracil in DNA is potentially mutagenic and a single unrepaired uracil opposite guanine can cause a C→T transition mutation upon replication, constituting a significant potential source of DNA damage. Genomic uracil can be introduced by deamination of cytidine, which occurs up to 500 times in a human cell each day [1]. Uracil in DNA can also arise as a result of enzymatic deamination of cytidine [2,3]. This is the obligatory initiating step in immunoglobulin (Ig) gene diversification, initiated by the B-cell specific enzyme, Activation Induced Deaminase (AID) [4-6]. In mammalian B cells, subsequent mutagenic processing of AID-initiated lesions results in somatic hypermutation and class switch recombination. Somatic hypermutation creates single base changes at the Ig variable (V) regions, providing a dynamic response to constantly mutating pathogens, and – when coupled with clonal selection – increased affinity of antibody for antigen. Class switch recombination deletes a large region of chromosomal DNA, rejoining DNA ends within G-rich switch (S) regions, and changing the constant region of the expressed Ig molecule without altering antigen specificity (reviewed by [2,7-9]).

Redundant pathways promote somatic hypermutation and class switch recombination in mammalian B cells [10,11]. One pathway depends upon uracil excision, carried out by uracil nucleoside glycosylase (UNG); and the other upon MutSα, a heterodimer of MSH2/MSH6. These factors also function in high fidelity repair pathways. In base excision repair, UNG excises uracil leaving an abasic (AP) site to be cleaved by AP endonuclease 1 (APE1), which is then repaired by pol β and DNA ligase 3 [1]. In the mismatch repair pathway, MutSα recognizes mismatches or damaged bases, hydrolyses ATP, recruits MutLα, promotes excision by Exonuclease I and then resynthesis by the high fidelity DNA polymerase δ (pol δ) in a reaction dependent upon proliferating cell nuclear antigen (PCNA) and replication protein A (RPA) (Reviewed by [12,13]).

The functional redundancy of the uracil excision and mismatch recognition pathways in Ig gene diversification raised the possibility that these pathways might also function redundantly in high fidelity repair of uracil in DNA. Sequence analysis of non-Ig genes in murine germinal center B cells has shown that AID acts widely, creating U•G mispairs which appear to be differentially subject to low-fidelity or high-fidelity correction [14]. Consistent with a potential role in repair of U•G mismatches, MutSα has been shown to specifically recognize heteroduplexes containing U•G *in vitro*, and to associate with targets of AID activity *in vivo *[15,16]. However, repair of U•G by the MutSα-provoked mismatch repair pathway to promote faithful correction of U•G mismatches has not been demonstrated.

We have now asked whether MutSα promotes repair at U•G mismatches *in vitro*. We show that purified human MutSα can recognize U•G but not U•A pairs; while, in contrast, UNG recognizes and removes uracil opposite either G or A. Heteroduplexes containing U•G pairs are efficiently corrected by MutSα; and like canonical mismatch repair of other base mismatches, efficient MutSα-dependent repair of U•G mismatches depends upon ATP, PCNA, and pol δ. Thus, redundant pathways carry out repair at UG, one pathway dependent upon UNG and the other upon MutSα. Faithful MutSα-dependent repair of heteroduplexes containing U•G mismatches occurs at comparable efficiency in extracts of B cells and non-B cells, suggesting that events or factors specific to the Ig loci of antigen-activated B cells must divert repair from a faithful to a mutagenic pathway. The efficiency of MutSα-directed repair of U•G is comparable to that of the UNG-directed pathway, and this may explain why, in humans, UNG-deficiency imparts compromised immunity but neither a decrease in genomic stability nor a broad predisposition to cancer.

## Results

### MutSα but not UNG Distinguishes U•G Mispairs from U•A Pairs in Duplex DNA

**Figure 1:**
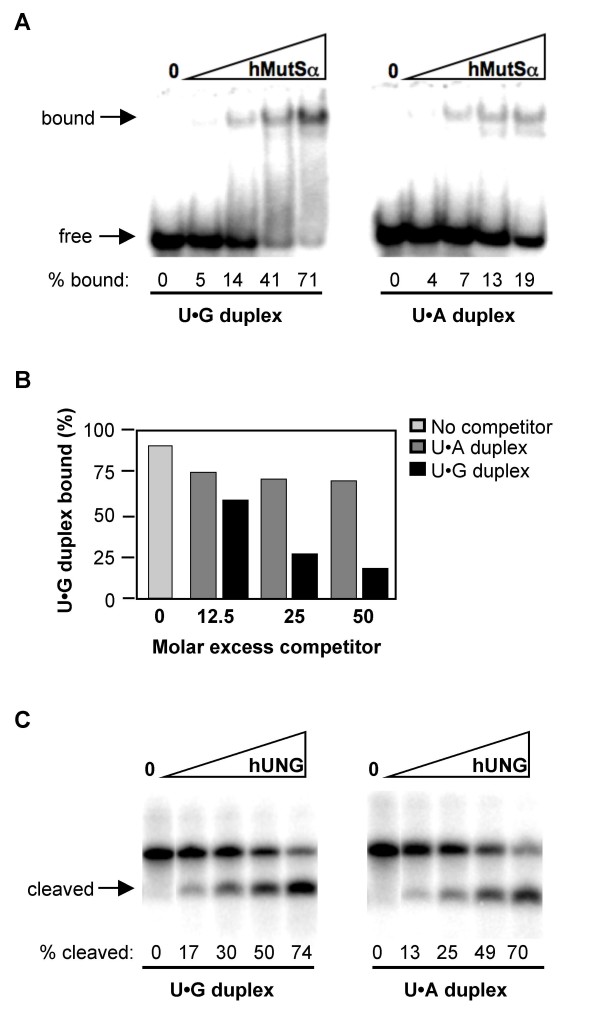
**MutSα but not UNG distinguishes U•G and U•A in duplex DNAs**. (A) Electrophoretic mobility shift assay of purified hMutSα (16, 32, 65 or 130 nM) binding to labeled DNA duplexes containing U•G mispairs (left) or U•A pairs (right). Arrows indicate bound and free DNA. The percentage of DNA bound is shown below. (B) Quantitation of binding of purified MutSα to radiolabeled U•G duplexes in the presence of indicated levels of unlabeled U•G or U•A duplex competitor. (C) Products of deglycosylation of DNA duplex substrates containing U•G mispairs or U•A pairs by 0, 0.2, 0.4, 0.8, or 1.6 nM of purified hUNG, resolved by denaturing gel electrophoresis. Substrates were 5' end labeled on the uracil-containing strand, and following incubation with hUNG were treated with alkali to hydrolyze the backbone at abasic sites. The fraction of cleaved molecules, quantified by phosphorimager, is shown below each lane.

We compared the binding of human MutSα (hMutSα) to synthetic DNA substrates containing U opposite either G (U•G) or A (U•A), using a gel mobility shift assay. hMutSα bound well to substrates containing U•G mismatches (apparent k_D _of 70 nM; Figure 1A), but poorly to substrates containing U•A pairs, (apparent k_D _> 135 nM). Competition analysis showed that binding of hMutSα to a labeled duplex substrate containing a single U•G mismatch was unaffected by the presence of a 50-fold molar excess of unlabeled duplex containing a U•A pair, but diminished more than 5-fold by a comparable amount of duplex DNA containing a U•G mismatch (Figure 1B). These properties are consistent with previous binding studies showing that hMutSα preferentially recognizes U•G or UU/GG heteroduplex oligonucleotides relative to homoduplexes [15-17]; and evidence that duplexes containing U•G but not U•A pairs activate MutSα ATPase activity [18].

Substrate preference of purified recombinant human UNG (hUNG) was analyzed by comparing deglycosylation activity on duplex substrates containing a U•G mispair or U•A pair. Deglycosylation creates an abasic (AP) site, which is alkali-labile, allowing ready quantitation of UNG activity on end-labeled substrates by treatment with alkali. hUNG had comparable activity on substrates containing U•A or U•G (Figure 1C). This is consistent with the documented ability of UNG to recognize unpaired uracil [19]; and with the ability of repair complexes associated with UNG to initiate short-patch base excision repair on DNA containing U opposite either G or A [20]. Thus, in the repair of genomic uracil, base excision repair responds to U•A or U•G while mismatch repair may specifically target U•G heteroduplexes.

### UNG-directed Repair of U•G Mispairs in Circular Duplex Substrates *in Vitro*

**Figure 2:**
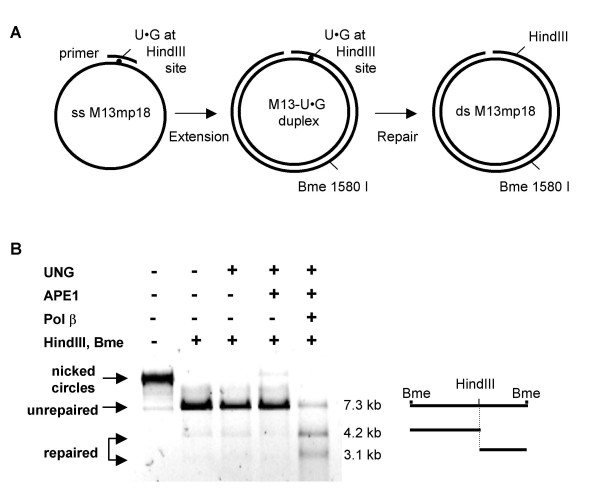
**UNG-directed repair of U•G mispairs in circular duplex substrates**. (A) Schematic for construction and repair of M13-U•G substrates. A 56-mer complementary oligonucleotide carrying a single U was annealed to ss M13mp18 to create a U•G mismatch within the *Hin*dIII restriction site (left); extended with Phusion polymerase to produce a nicked-circular duplex M13-U•G substrates (center); and repaired to create a functional *Hin*dIII site (right). (B) Reactions demonstrating repair of M13-U•G substrates by purified components of the base excision repair pathway, analyzed by agarose gel electrophoresis. Shown are unrepaired, undigested M13-U•G substrates; and substrates treated with UNG, APE1 and pol β and then digested with *Hin*dIII and *Bme *1580 I, as indicated. Arrows at left indicate undigested nicked circles, and unrepaired and repaired products; schematic at right diagrams products of *Hin*dIII and *Bme *1580 I digestion.

Specific recognition by hMutSα of DNA duplexes containing U•G mispairs (Figure 1) suggested that, in human cells, genomic uracil is corrected by MutSα-directed mismatch repair pathway. To test this possibility, we developed circular ds DNA substrates containing a single U at a defined position, which could be used to assay repair by either pathway. These substrates were modeled on those used to define the mechanism of MutSα-driven repair at single-base mismatches and chemically modified bases, which contain a mismatched or damaged base at a defined position and a single-strand DNA discontinuity (nick or short gap) to direct repair to the strand containing the damage [21-27]. A synthetic oligonucleotide carrying a U within the single *Hin*dIII restriction site in the *lacZα *gene was annealed to single-stranded circular M13mp18 DNA (Figure 2A, left); and then extended with the high-fidelity Phusion polymerase (New England Biolabs), which lacks both 5'-3' exonuclease and strand displacement activity. This created a duplex circular molecule, containing a single U•G mismatch that interrupts the *Hin*dIII restriction site, and a strand discontinuity (nick or short gap) 38 nt from the mismatch (Figure 2A, center). This substrate, referred to henceforth as M13-U•G, cannot be cleaved by *Hin*dIII, which requires the correct palindromic sequence (AAGCTT) on both DNA strands. The strand discontinuity will direct mismatch repair to the strand containing the single U, and repair of U•G to C•G will create a site cleavable by *Hin*dIII (Figure 2A, right). Substrates contain a single *Bme *1580 I site remote from the mismatch. Simultaneous digestion of the unrepaired substrate with *Bme *1580 I and *Hin*dIII will create a single linear fragment 7.3 kb in length; while digestion of repaired DNA will generate two fragments, of 4.2 and 3.1 kb. The ratio of these two fragments to total DNA provides a measure of efficiency of repair.

We confirmed that the M13-U•G ds substrates could report on repair by the UNG-dependent base excision pathway by assaying reconstitution of the *Hin*dIII site after sequential incubation with purified hUNG, hAPE1, and DNA polymerase β (pol β), members of the UNG-directed base excision repair pathway. *Hin*dIII was unable to cleave M13-U•G following incubation with UNG, or UNG and APE1; however, subsequent incubation with pol β resulted in 68% cleavage by *Hin*dIII (Figure 2B). Therefore, the M13-U•G circular substrates sensitively monitor reconstitution of the *Hin*dIII site by *in vitro *repair.

### UNG-independent Repair of U•G Mispairs in Human Nuclear Extracts

**Figure 3:**
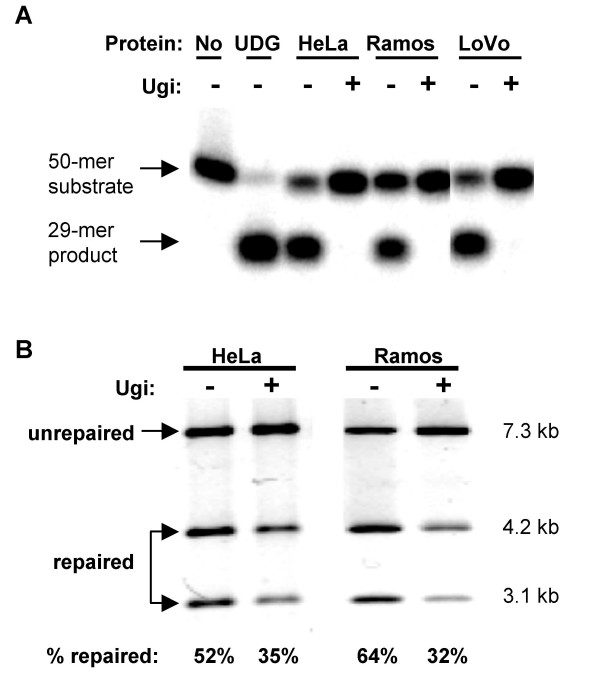
**UNG directs repair of only a fraction of U•G mispairs in human nuclear extracts**. (A) Products of deglycosylation of a 5'-end-labeled single-stranded oligonucleotide containing a single uracil, following incubation with recombinant UDG or nuclear extracts prepared from HeLa, Ramos, and LoVo cell lines in the presence (+) or absence (-) of Ugi. Substrates were treated with alkali following incubation to hydrolyze the backbone at abasic sites. Arrows indicate substrate and product. (B) Representative reactions demonstrating repair of M13-U•G substrates by nuclear extracts of HeLa or Ramos, in the presence (+) or absence (-) of Ugi, analyzed by agarose gel electrophoresis. Arrows at left indicate unrepaired and repaired products; fragment sizes are shown at right; fraction of molecules repaired is shown below each lane.

To distinguish the contributions of MutSα and UNG to repair of U•G mismatches, we first asked how inhibition of UNG affected repair of M13-U•G circular substrates in human cell extracts. The small protein, uracil glycosylase inhibitor (Ugi), encoded by bacteriophage PBS2, is specific for UNG homologs and forms a 1:1 complex with the DNA binding domain of UNG, blocking uracil excision *in vitro *and *in vivo *[28-31]. We prepared nuclear extracts from three human cell lines: HeLa, derived from an adenocarcinoma [22,32]; Ramos, derived from an actively hypermutating B cell lymphoma [33]; and LoVo, derived from an MSH2-deficient colorectal carcinoma [34]. All three extracts showed high levels of uracil DNA glycosylase activity; and Ugi completely inhibited deglycosylation in all three extracts (Figure 3A). There are other uracil DNA glycosylases in human cells, including MBD4, TDG, and SMUG1, which are divergent from UNG [35] and therefore should be insensitive to Ugi. The fact that uracil DNA glycosylase activity could not be observed in the presence of Ugi confirms that UNG is the predominant activity for deglycosylation of uracil in these extracts; and shows that treatment of human nuclear extracts with Ugi can be used to distinguish UNG-directed base excision repair from UNG-independent repair.

We then assayed repair of M13-U•G circular substrates in nuclear extracts prepared from HeLa and the constitutively hypermutating B cell line, Ramos, in the presence or absence of Ugi. HeLa nuclear extracts supported repair of 52% of the molecules containing U•G mismatches, and this diminished to 35% in the presence of Ugi (Figure 3B). Ramos supported repair of 64% of the molecules containing U•G mismatches, and this diminished to 32% in the presence of Ugi (Figure 3B). Repair values in the absence of Ugi are consistent with published values for correction of single base mismatches by human mismatch repair in vitro [22-25,32]. U•G mismatch repair in Ugi-treated extracts was reproducible. Repair efficiency of U•G in three independent experiments averaged 33% repair (S.E. 1.76%) in Ugi-treated HeLa extracts; and 32% (S.E. 1.45%) in Ugi-treated Ramos extracts. Thus, Ugi-sensitive, UNG-directed repair accounts for 30–50% of repair of U•G mispairs in Ramos or Hela extracts, while the remainder (and major fraction) of repair is Ugi-insensitive and UNG-independent. Because Ugi blocked detectable uracil glycosylase activity in HeLa and Ramos nuclear extracts (Figure 3A), Ugi-insensitive repair must be carried out by activities independent of base excision repair.

### MutSα and UNG Provide Redundant Pathways for Repair of U•G Mispairs

**Figure 4:**
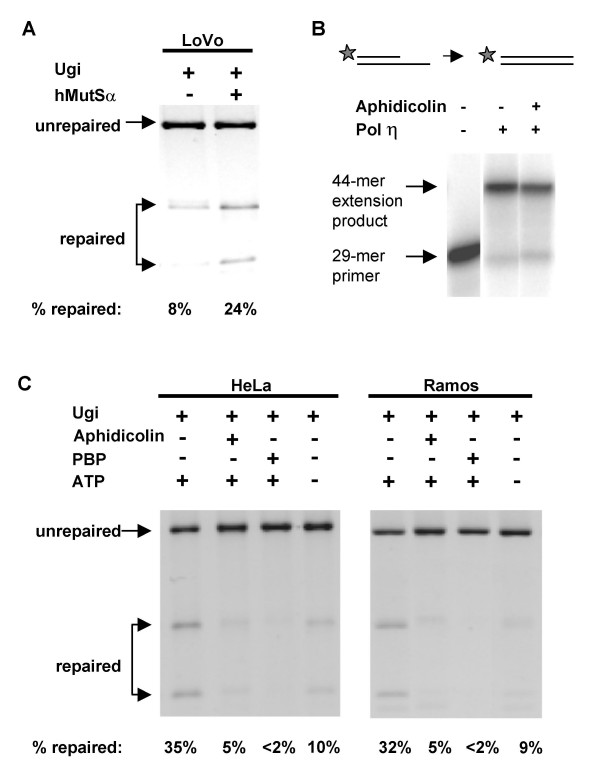
**MutSα directs faithful repair of U•G mispairs in human nuclear extracts**. (A) Representative reactions demonstrating repair of M13-U•G substrates by Ugi-treated LoVo nuclear extracts, in the absence (-) or presence (+) of 53 nM purified hMutSα. Arrows at left indicate unrepaired and repaired products; fraction of molecules repaired is shown below each lane. (B) Products of extension by pol η in the absence or presence of aphidicolin. Arrows at left indicate primer and extension product. (C) Representative reactions demonstrating repair of M13-U•G substrates by Ugi-treated HeLa or Ramos nuclear extracts, in the presence and absence of aphidicolin, which inhibits pol δ; PBP, the PCNA binding peptide, which inhibits PCNA; or exogenous ATP. Notations as in panel A.

To ask if MutSα can direct repair of U•G mispairs, we assayed repair of the M13-U•G circular substrates in Ugi-treated extracts derived from the MSH2-deficient cell line LoVo. Mismatch repair is defective in LoVo extracts but can be restored *in vitro *by addition of purified hMutSα [23,24,32,36,37]. Nuclear extracts of LoVo, treated with Ugi to inhibit UNG (e.g. Figure 3A), supported repair of only 8% of the M13-U•G substrates (Figure 4A). Addition of purified hMutSα to Ugi-treated LoVo nuclear extracts increased repair 3-fold, to 24% (Figure 4A). Thus, MutSα promotes efficient repair of U•G mispairs.

We then asked if MutSα-dependent repair of U•G mispairs requires pol δ, ATP, and PCNA, all essential for canonical mismatch repair *in vitro *[38-40]. Pol δ activity and mismatch repair are both blocked by aphidicolin [38,41]. In a control experiment, we established that aphidicolin does not block the mutagenic polymerase pol η, which is strongly implicated in MutSα-directed hypermutation [42,43], by showing that aphidicolin did not affect extension by pol η of a 5'-end-labeled 29-mer oligonucleotide annealed to a 50-mer template to produce a predicted 44-mer duplex extension product (Figure 4B). However, aphidicolin almost completely prevented repair of M13-U•G mispair substrates in Ugi-treated HeLa and Ramos nuclear extracts, diminishing repair to 5%, from 35% (HeLa) and 32% in Ramos (Figure 4C). This shows that pol δ participates in MutSα-directed repair of U•G mispairs. The low level of repair evident in Ugi-treated nuclear extracts in the presence of aphidicolin may be due to participation of aphidicolin-resistant polymerases.

PCNA binding protein (PBP) inhibits excision and DNA resynthesis during mismatch repair by blocking PCNA function [38,40]. Repair of M13-U•G mispair substrates was essentially abolished when PBP was added to Ugi-treated nuclear extracts (Figure 4C). Thus, UNG-independent repair of U•G mispairs depends upon PCNA.

Omission of ATP caused the percentage of molecules repaired to decrease from 35% to 10% in Ugi-treated HeLa nuclear extracts; and from 32% to 9% in Ugi-treated Ramos nuclear extracts (Figure 4C). Low levels of ATP in the extracts may account for repair in the absence of exogenous ATP.

The dependence of repair of M13-U•G substrates on MutSα, PCNA, pol δ, and ATP, establishes that the canonical mismatch repair pathway can correct U•G mismatches in duplex DNA molecules.

### High Fidelity Repair of U•G Mispairs in Extracts of a Hypermutating Human B Cell Line

**Figure 5:**
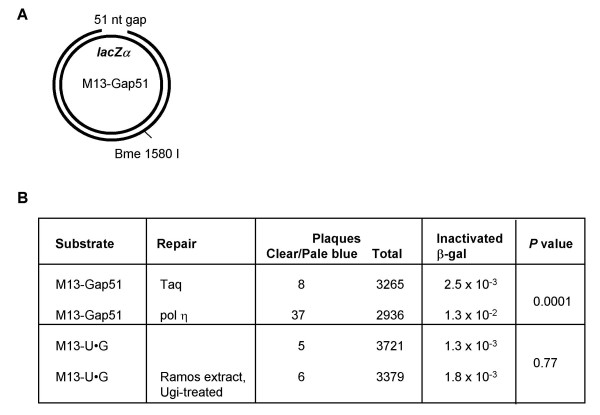
**Nuclear extracts from the hypermutating B cell line, Ramos, support faithful repair of U•G mispairs**. (A) Schematic of M13-Gap51 substrates, which carry a 51 nt gap in the *lacZα *region of M13mp18. (B) Efficiency of repair of M13-Gap51 or M13-U•G substrates, determined by quantitating the fraction of clear and pale blue plaques on X-gal indicator plates. *P *values were determined by two-tailed Fisher's exact test.

Comparable levels of U•G repair were observed in nuclear extracts of HeLa and the hypermutating B cell line, Ramos (Figures 3 and 4), suggesting that hypermutation does not reflect compromised function of faithful repair pathways. To further characterize repair fidelity, we used an assay that measures inactivation of β-galactosidase due to mutagenesis that occurs during synthesis across a gap in the *lacZα *gene in M13mp18 phage, quantitating the fraction of clear or pale blue plaques produced upon transformation of *E. coli *followed by plating on a helper lawn on X-gal plates [44]. This assay provides convenient comparison of mutation frequencies under different conditions, but underestimates total mutation frequency because not all mutations affect β-galactosidase activity. As a positive control, we used the M13-Gap51 substrate, in which a 51 nt gap, extending from the site of the mismatch at the *Hin*dIII site (position 6281) to the *Eco*RI site (position 6230) near the 5' end of *lacZα *(Figure 5A), recapitulates the short excision tract created during mismatch repair of the M13-U•G substrate. We compared mutagenesis following incubation of M13-Gap51 substrates with either the faithful polymerase, Taq, or the mutagenic polymerase, pol η, to fill the gap. The proportion of clear or pale blue plaques was 2.5 × 10^-3 ^following by gap filling by Taq, and 1.3 × 10^-2 ^following gap filling by pol η (Figure 5B). These numbers are significantly different (*P *= 0.001, two-tailed Fisher's exact test), confirming that this assay provides a sensitive measure of mutagenesis.

We used the same assay to determine the frequency of β-galactosidase inactivation in the M13-U•G substrates. The background frequency of β-galactosidase inactivation evident upon direct transformation of *E. coli *with unrepaired M13-U•G substrates was 1.3 × 10^-3 ^(Figure 5B). When M13-U•G substrates were incubated in Ugi-treated Ramos extracts to allow repair to occur prior to transformation, the proportion of clear or pale blue plaques was 1.8 × 10^-3^, not significantly different from unrepaired M13-U•G substrates (P = 0.77, two-tailed Fisher's exact test; Figure 5B). Parallel analysis showed that the fraction of repaired substrates, as assayed by *Hin*dIII-sensitivity, was 34%, comparable to the efficiency of repair in Ugi-treated Ramos nuclear extracts (not shown). Thus, the extracts were active for repair, but mutagenesis appeared not to accompany repair.

The plaque color-based mutagenesis assay scores only mutations that inactivate β-galactosidase. To ensure that this assay did not greatly underestimate the mutation frequency, we sequenced randomly selected blue plaques arising from M13-U•G incubated in the repair extract prior to transformation of *E. coli*. A majority of the excision tracts initiated by *in vitro *mismatch repair of nick-circular substrates terminate between 90 and 170 bases from the mismatch [45]. We therefore analyzed a 200 nt region bordered by the strand discontinuity and encompassing DNA upstream of the mismatch site (Figure 2A) for mutations induced in the course of nick-directed uracil repair. Within this region, we did not find significant mutagenesis of UG-M13 substrates incubated with Ugi-treated HeLa extract (57 plaques, no mutations, 11,400 nt sequenced) or from Ramos nuclear extract (93 plaques, 1 mutation, 18,600 nt sequenced). Thus, in contrast to pol η-directed gap filling, repair of U•G by the human nuclear extracts was not mutagenic.

## Discussion

We have shown that multiple repair pathways may correct genomic uracil. This is consistent with published evidence showing that deficiency in UNG activity in humans is most evident as immunodeficiency, rather than as predisposition to malignancies [46,47]. Similarly, disruption of the murine *Ung *gene did not result in greatly increased levels of spontaneous mutations [48], although it did cause immunodeficiency [11]. This has been ascribed to the presence of redundant factors which can deglycosylate uracil, such as SMUG1, TDG, or MBD4 [35]. Our results now suggest that MutSα may provide an equally important pathway for faithful repair of uracil in DNA.

These results expand the scope of substrates known to be repaired by hMutSα to include U•G heteroduplexes. hMutSα interacts nonspecifically with homoduplex DNA [49], but specifically recognizes a broad array of substrates, including single-base mismatches and small loops [21,36]; base alterations such as 8-oxoguanine paired opposite thymine [23,49]; O^6^-alkylguanine and cisplatinated guanine [50], UV photoproducts [17]; and also alternatively structured DNA, such as Holliday junctions and G4 DNA [15,51].

The UNG-directed and MutSα-directed U•G repair pathways were comparably efficient in the actively hypermutating B cell line, Ramos, and in HeLa cells. The evidence that U•G repair is not intrinsically mutagenic in B cells suggests that a localized mechanism may divert repair at diversifying Ig genes, enabling mutagenic pathways to co-opt normally faithful repair pathways. Hypermutating Ig V regions exhibit enrichment of the MRN complex and associated MRE11 AP lyase activity, but not of APE1; and cleavage by MRE11 rather than APE1 may divert repair to low-fidelity pathways [52]. MutSα interacts with pol η [16], which could promote low fidelity repair in the course of resynthesis through the excision tract [2,42]. Ubiquitination of PCNA provides one possible mechanism for regulating the switch between pol δ and pol η [53].

The Ig gene diversification apparatus takes advantage of differences between the UNG-directed and MutSα-directed repair pathways to ensure that mutations spread beyond the C/G pairs, the site of AID activity. In faithful repair MutSα recruits Exonuclease I, which promotes excision from a nick through the region containing the mismatch. In Ig gene hypermutation, MutSα promotes mutagenesis at A/T pairs, which are not AID targets, in a process dependent upon both the MSH2 ATPase [54] and Exonuclease I [55]. In contrast, at hypermutating Ig genes UNG promotes mutagenesis at C/G pairs, the sites of AID-initiated DNA damage [56], recapitulating the very limited excision and resynthesis of a single nucleotide typical of faithful repair directed by UNG, APE1 and DNA polymerase β [1].

It is not yet known whether strand-specificity governs MutSα-dependent events at the Ig genes of diversifying B cells, or the time in the cell cycle that diversification occurs. Nonetheless, it is important to note that if MutSα directs post-replicative repair, and if the uracil in a U•G mispair is located on the parental strand, function of MutSα would result in a G→A transition mutation, not correcting but "fixing" the mutation. A similar paradoxical situation has been noted for MutSα-directed repair of parental-strand base damage, such as 8-oxouanine [23], O^6^-alkylguanine [27,57] and UV photoproducts [17]. Diversifying Ig genes could capitalize on this apparently paradoxical function to increase the level of mutagenesis, by regulating AID attack and participation of MutSα within cell cycle. Future experiments should address this possibility.

## Conclusion

We have directly demonstrated by biochemical assays that human MutSα can direct faithful correction of U•G mismatched DNA via the canonical mismatch repair pathway. These results identify redundant roles for mismatch repair and base excision repair in correction of genomic uracil in the cell. Moreover, they show that participation of these pathways in immunoglobulin gene diversification reflect their redundant functions in faithful repair.

## Methods

### Proteins and Nuclear Extracts

Human UNG2 (hUNG) was PCR amplified from cDNA from the Ramos cell line using primers 5'- CACCATGATCGGCCAGAAGACGCTCTACTCCTTTTTCTC and 5'- TCACAGCTCCTTCCAGTCAATGGGCTTCTTGCC; and cloned downstream of the T7 promoter in pET100 (Invitrogen, Carlsbad, CA), providing an N-terminal His_6 _tag. Following sequence confirmation, the hUNG plasmid was used to transform *E. coli *BL21 DE3 pLysS (Invitrogen), in which T7 RNA polymerase is expressed under control of the lactose repressor. Mid-log cells were cultured at 37°C for 2 hr with 1 mM IPTG to induce T7 polymerase expression; collected by centrifugation; frozen; resuspended in 50 ml of lysis buffer containing 20 mM Tris pH 7.7, 300 mM NaCl, 0.5 mM DTT, and a protease inhibitor mini-tablet (Roche, Indianapolis, IN); sonicated twice for 20 seconds, and cell debris removed by centrifugation. hUNG was purified by Ni^++ ^chromatography (Sigma, St. Louis, MO) according to manufacturer's instructions, followed by S-Sepharose gravity flow chromatography (GE, Piscataway, NJ). hAPE1 was purified as described previously [52]. Pol β was purchased from Trevigen (Gaithersburg, MD). MutSα was overexpressed by co-infecting Sf9 insect cells with baculovirus vectors expressing MSH2 and MSH6 and purified as previously described [15]. HeLa, Ramos, and LoVo cells were supplied by the National Cell Culture Center (Minneapolis, MN) as pellets stored on wet ice, and nuclear extracts were prepared as previously described [37]. Protein concentrations were determined using BSA as a standard.

### DNA Substrates

Synthetic duplexes carrying U•G mismatches were created by annealing EDL56, 5'-GCCGAATTTCTAGAATUGAAAGCTTGCTAG-3', to EDL364, 5'-CTAGCAAGCTTTCGATTCTAGAAATTCGGC-3'; U•A duplexes were generated by annealing EDL56 to EDL365, 5'-CTAGCAAGCTTTCAATTCTAGAAATTCGGC-3'; and homoduplexes were generated by annealing EDL365 to EDL366, 5'- GCCGAATTTCTAGAATTGAAAGCTTGCTAG-3'. Oligonucleotides were 5'-end labeled using T4 PNK (NEB, Ipswich, MA) and γ-^32^P-ATP. To generate labeled duplexes, EDL56 was 5'-end-labeled prior to annealing. A single-stranded 50-mer oligonucleotide, 5'-CAGAAAGGGAAAGTATACAACAAAAAGCAUCTCAAGTCTTGGAGAGAACA, was used to assay hUNG activity in nuclear extracts. Extension assays using pol η were primed from 5' end labeled EDL145, 5'-CAGAAAGGGAAAGTATACAACAAAAAGCA-3' annealed to EDL119, 5'- CTCCAAGACTTGAGGTGCTTTTTGTTGTATACTTTCCCTTTCTGTGACCT-3'.

M13-U•G double-stranded (ds) circular substrates for mismatch repair assays were designed to carry a single U•G mismatch within a *Hin*dIII restriction site, and a 5' strand discontinuity (nick or short gap) on the strand targeted for repair [22,25,58]. M13mp18 circular single-stranded (ss) (+) strand DNA (NEB) was annealed to PAGE purified oligonucleotide EDL330, 5'-CCCAGTCACGACGTTGTAAAACGACGGCCAGTGCCAAGUTTGCATGCCTGCAGGTC, in a 1.4 ml reaction that contained 13.7 μg M13mp18 DNA, 80 pmol EDL330, 0.2 mM dNTPs, in Phusion polymerase HF buffer (NEB), to create a U•G mismatch at position 6283 (underlined); extended with Phusion polymerase (14 U) at 65°C for 45 min; and duplex extension products separated from other reaction components by two rounds of purification on PCR-pure spin columns (Qiagen, Valencia, CA). To ensure that no homoduplex molecules were present (which could in principle be produced by strand displacement, although this is not a reported activity of Phusion polymerase), products were digested with *Hin*dIII (1 U/μg substrate); single-stranded M13mp18 was removed by BND cellulose chromatography (Sigma, St Louis, MO); and linear molecules destroyed by treatment with Exonuclease V (USB, Cleveland, OH). Following ethanol precipitation, M13-U•G ds circles were shown to co-migrate with nicked, duplex M13mp18 upon 1% agarose gel electrophoresis (420 V•hr).

### Repair Activity and Binding Assays

Uracil repair and binding assays results were confirmed by repetition and representative results are presented. Images were captured and analyzed by phosphorimager (GE) or captured and quantified by an AlphaInnotech HD2 system and presented as reversed ethidium bromide stained images as indicated for each assay.

MutSα binding assays were performed in 30 μl reactions containing 25 mM Tris pH 7.7, 100 mM KCl, 1 mM EDTA, 1 mM DTT, 7.5% glycerol, with indicated concentrations of purified MutSα, 10 fmol radiolabeled heteroduplex, and indicated concentrations of homoduplex oligonucleotide competitor DNA (annealed EDL365/EDL366). Following incubation on ice for 5 min, reactions were brought to 0.7% Ficoll, and then resolved by 6% native PAGE in 0.5 × TBE. For competition experiments, indicated concentrations of unlabeled U•G (EDL56/EDL364) or U•A (EDL56/EDL365) duplexes were added prior to addition of protein.

UNG activity was assayed by quantitating production of the alkaline-labile abasic site generated upon deglycosylation of uracil. Purified recombinant hUNG (40 to 2.5 fmols) and 50 fmols of U•G (EDL56/EDL364) or U•A (EDL56/EDL365) synthetic duplexes were incubated in 25 μl reactions containing 10 mM Tris, 1 mM EDTA, 37°C for 15 min; treated with 0.25% SDS, 10 mM EDTA and 100 μg/ml Proteinase K (Promega, Madison, WI) at 37°C for 10 min; and then with 0.1 N NaOH at 37°C for 5 min. Following addition of an equal volume of formamide, cleavage products were resolved by 15% denaturing PAGE. Images were captured and quantified by phosphorimager. UNG activity present in human nuclear extracts was determined by a similar procedure, using 50 μg of nuclear extract and 50 fmols of 5'-end-labeled EDL58 oligonucleotide in 30 μl reactions containing 20 mM Tris pH 7.7, 100 mM KCl, 5 mM MgCl_2_, 1 mM glutathione, 1.5 mM ATP, 0.1 mM each dNTP, and 50 μg/ml BSA for 20 min at 37°C. Ugi (NEB, 2 U in 1 μl) was added to inhibit UNG.

Mismatch repair assays followed a published protocol [22]. M13-U•G ds circular substrates (13.6 fmol) were incubated with 50 μg of nuclear extract in 30 μl reactions containing 20 mM Tris pH 7.7, 100 mM KCl, 5 mM MgCl_2_, 1 mM glutathione, 1.5 mM ATP, 0.1 mM each dNTP, and 50 μg/ml BSA for 20 min at 37°C. In some cases, recombinant human MutSα (hMutSα) was added (1.6 pmol), or pol δ was inhibited with aphidicolin (183 μM; Sigma); or PCNA was inhibited by addition of PCNA binding protein (PBP, American Peptide Company Sunnyvale, CA), as described [24]. Reactions were terminated by addition of 15 μl (one-half volume) 1% SDS, 25 mM EDTA pH 8, 0.1 mg/ml Proteinase K; and following phenol extraction and ethanol precipitation, DNA was resuspended in 1× NEB Buffer 2 supplemented with 100 μg/ml RNAse A, 100 μg/ml BSA. DNA was then cleaved by simultaneous digestion with *Bme *1580 I (1 U), which linearizes M13mp18 at position 2088; and *Hin*dIII (1 U), which cleaves only those molecules in which the U•G mismatch has been converted to a C•G pair in the course of DNA resynthesis, creating a *Hin*dIII site (AAGCTT) at position 6281. Digestion products were resolved by 1% agarose gel electrophoresis, gels stained with ethidium bromide, and images captured and quantified using an AlphaInnotech HD2 imaging system. Representative images are presented as reversed ethidium bromide stained images.

### Pol η Extension Assay

The activity pol η was measured by extension of 5'-^32^P-labeled oligonucleotide EDL145, annealed to EDL119, with 160 fmols purified recombinant pol η (Enzymax, Lexington, KY), in buffer conditions identical to those used for mismatch repair assays (20 mM Tris pH 7.7, 100 mM KCl, 5 mM MgCl_2 _1 mM glutathione, 1.5 mM ATP, 0.1 mM each dNTP, and 50 μg/ml BSA), in the presence or absence of 180 μM aphidicolin. The 44-mer extension product was distinguished from the EDL145 29-mer primer by 15% denaturing PAGE.

### Repair Fidelity Assay

The assay for fidelity of U•G heteroduplex repair was based on an assay developed for quantitating DNA polymerase fidelity on gapped M13mp2 molecules [44]. Control M13-Gap51 substrates, containing a 51 nt gap within the *lacZα *gene of M13mp18, were generated by annealing a 10-fold molar excess of M13mp18 ssDNA circles to a slightly shorter complementary strand, produced by digestion of duplex M13 with *Eco*R1 and *Hin*dIII, and removal of the 51 bp fragment using a PCR purification column (Qiagen). Unpaired linear single strands and any circular single-stranded M13mp18 were removed by BND cellulose chromatography and duplex linear DNA was removed by digestion with Exonuclease V (USB). The gap in M13-Gap51 was filled with either Taq polymerase (1 U, NEB) or 160 fmols pol η (12.5 ng), under manufacturer's standard conditions. Repair was carried out in 30 μl reactions in the same conditions as the mismatch repair assay (above). Recovered DNA was resuspended in 30 μl TE, and repair at the *Hin*dIII site was quantitated by digestion of a 5 μl aliquot with *Bme *1580 I and *Hin*dIII followed by gel electrophoresis, as described above. Repair fidelity was analyzed by a plaque assay for β-galactosidase activity, in which *E. coli *MC1061 was transformed with 1.4 ng of DNA by electroporation, transformants plated with the helper strain CSH50, on minimal glucose media containing X-gal (Sigma) and unmutated (blue), or mutated (pale blue or clear) plaques quantitated. *E. coli *strains were provided by Dr. Lawrence A. Loeb, University of Washington, Seattle, WA.

## Abbreviations

MSH: Mut S Homolog; UNG: Uracil Nucleoside Glycosylase; Ugi: Uracil Glycosylase Inhibitor; PCNA: Proliferating Cell Nuclear Antigen; AID: Activation Induced Deaminase.

## Authors' contributions

Conceived and designed the experiments: EDL, NM. Performed the experiments: EDL, DWB. Analyzed the data: EDL, DWB, NM. Wrote the paper: EDL, NM.
